# Prevalence of anemia among Lebanese hospitalized children: Risk and protective factors

**DOI:** 10.1371/journal.pone.0201806

**Published:** 2018-08-07

**Authors:** Ali Salami, Hisham F. Bahmad, Ghassan Ghssein, Lamis Salloum, Hadi Fakih

**Affiliations:** 1 Department of Mathematics, Faculty of Sciences (V), Lebanese University, Nabatieh, Lebanon; 2 Department of Anatomy, Cell Biology, and Physiological Sciences, Faculty of Medicine, American University of Beirut, Beirut, Lebanon; 3 Department of Biology, Faculty of Sciences (V), Lebanese University, Nabatieh, Lebanon; 4 Pediatrics Department, Ragheb Harb Hospital, Iranian Red Crescent, Toul, Lebanon; 5 Pediatrics Department, Faculty of Medical Sciences, Lebanese University, Beirut, Lebanon; Istituto di Ricovero e Cura a Carattere Scientifico Centro di Riferimento Oncologico della Basilicata, ITALY

## Abstract

**Background:**

Anemia is a global health problem associated with short- and long-term consequences especially in children. The incidence of anemia along with the factors associated with its increased or decreased risk is not yet well studied in Lebanon. Our study aims at determining the demographics of this health burden and identifying some of the important factors linked to it among the pediatric population.

**Methods:**

A 4-months cross-sectional study was performed between August and November 2017 including 295 children aged 1 month to 12 years, who were hospitalized in a tertiary care hospital located in South Lebanon. We analyzed the different demographic data, age, gender, breast feeding duration, solid food introduction, iron supplementation and disease of diagnosis in association with multiple hematological parameters.

**Results:**

The prevalence of both mild and moderate anemia was 71.8 and 25.4%, with only 2 cases of severe anemia encountered among children aged 6 months or above. Results showed that the risk of anemia increases by around 3.4 folds among malnourished children than in well-nourished children. This risk also decreased by almost 42% in children receiving iron supplement.

**Conclusion:**

In consideration to the fact that anemia is a prevalent disease in the Lebanese childhood population, especially in infancy, simple preventive measures such as proper nutritional habits and supplementation of iron rich food to children are highly recommended and should be respected by public health providers.

## Introduction

Worldwide, one of the major public health concerns is anemia, especially among children. Identifying its major causes and implementing proper interventions are highly demanded [1]. As estimated by the World Health Organization (WHO), about 1.62 billion people suffer from anemia globally, out of which approximately 50% affect preschool-age children (highest prevalence) [2–4]. An updated data about the global estimate of childhood anemia indicates that, worldwide, around 43% of children aged below five years have anemia, among which 28.5% live in sub-Saharan Africa [5, 6]. Iron deficiency and its associated anemia are more common among children, those on poor diets, people with intestinal parasitic diseases, and women of childbearing age [5].

Anemia is a multifactorial clinical disease contributed to inappropriate nutritional habits and poor supplementation of essential factors involved in the production of red blood cells, some parasitic infestations, chronic inflammatory diseases, genetic predisposition, etc [7]. Parasitic infections are more common in developing countries and frequently instigate microcytic anemias via iron deficiency and depletion that is mainly attributed to the blood loss from gastrointestinal infections [8, 9].

Anemia is defined as a reduction in red blood cell (RBC) mass or blood hemoglobin (Hgb) concentration. Yet, clinically, physicians consider anemia whenever there are reductions in one or both of the following: hematocrit (Hct) and Hgb, the latter of which is a subrogate value for anemia [10]. However, the RBC count may be misleading in the evaluation of anemia, where in some cases of microcytic anemia, thalassemia for instance, there is usually an elevated erythrocyte count [9].

As a consequence of anemia, a status of low brain tissue oxygenation is encountered that may cause, especially in children, cognitive function impairment, and affects psychomotor development and physical growth [11]. Due to their rapid body growth and their high RBCs expansion, children below five years of age have increased iron needs, thus are more susceptible to develop anemia [12].

Anemia has many predisposing factors that include socioeconomic and environmental conditions in addition to other biological and nutritional factors. Available data concerning the prevalence of anemia and associated cofactors and implicating conditions in Lebanon are scarce, thus this study aimed to determine the prevalence of anemia in a hospitalized pediatric population (up to 12 years of age) admitted to a tertiary care hospital located in South Lebanon.

## Materials and methods

### Patients selection

Parents of all patients enrolled in this cross sectional clinical study provided written informed consents for both participation and publication of identifying information, in accordance with the Declaration of Helsinki. The study with all its experimental protocols was conducted under the Institutional Review Board (IRB) approval of the Lebanese University (LU) and the Ethics Committee of Ragheb Harb Hospital (RHH). Ethical clearance was taken as per the norms and in accordance with relevant guidelines and regulations of RHH and LU. Recruitment was done randomly after obtaining a written informed consent from the patient care givers.

Clinical data from 295 pediatric patients, aged from 1 month old up to 12 years old and admitted to RHH between August and November 2017, were enrolled in this cross-sectional study to assess the demographic and predisposing factors as well as appropriate preventive measures associated with anemia. Ragheb Harb Hospital (RHH) is a tertiary care medical center located in the District of Nabatieh, South Lebanon, that serves a population of around 350,000 habitants, with an approximate annual admission of 5,000 children to the pediatric department, the latter which includes 35 beds.

Children were excluded if they have an active hemorrhage, bleeding disorders, history of blood transfusion and or any immunodeficiency.

### Clinical variables

Clinical data pertaining to each patient, including gender, age, weight, serum hemoglobin concentration, type of feeding and nutrition, and disease of diagnosis prompting hospitalization, were collected for all patients. Patients were categorized by age as follows: 1 month, 2 months, 3–5 months, 6–59 months, and more than 60 months (up to 12 years).

Routine procedures of the hospital were followed to measure hemoglobin concentrations, red blood cell counts, and mean corpuscular volume (MCV), with levels being determined using the Sysmex xn-3000 apparatus (Sysmex Europe GmbH, Germany). As per the World Health Organization (WHO) and American Academy of Pediatrics (AAP) criteria, anemia is defined as Hgb concentrations below 2.5^th^ percentile for age, race and gender [3, 10]. In children aged 1, 2, and 3–5 months, the cutoff Hgb value for anemia is set at 10.7, 9.4, and 9.5 g/dL respectively; in children aged 6 to 59 months, the cutoff Hgb value for anemia is set at 11g/dL; and in older children aged 60 months to less than 12 years, the cutoff value is 11.5g/dL [13–16]. This definition was considered to stratify our patients into anemic and non-anemic groups (**Tables 1 and 2**). Generally, results of the first blood test performed at time of admission were considered for most of our patient. Anemia was further categorized in children aged 6 to 59 months as mild (Hgb = 10.0–10.9 g/dL), moderate (Hgb = 7.0 to 9.9 g/dL), and severe (Hgb < 7.0 g/dL), and in children aged 5 to 14 years as mild (Hgb = 11.0–11.4 g/dL), moderate (Hgb = 8.0 to 10.9 g/dL), and severe (Hgb < 8.0 g/dL) [1, 17] (**Table 3**).

**Table 1:** Socio-demographic and clinical characteristics of 295 pediatric patients enrolled in the study.

Clinical parameter	Total number of patients	Categories	N	%	P-value
**Gender**	295	Males	160	54.2	0.146
		Females	135	45.8	
**Age (months)**	295	1 month	7	2.4	**<0.001**
		2 months	16	5.4	
		3–5 months	12	4.1	
		6–59 months	212	71.9	
		≥60 months to <12 years	48	16.3	
Anemia status*	292	Anemic	81	27.7	**<0.001**
		Non-anemic	211	72.3	
**Percentage of anemia according to gender**	81	Male	45	55.6	0.759
		Female	36	44.4	
**Severity of anemia (g/dL) in children 0.5–12 years**	71	Mild	51	71.8	**<0.001**
		Moderate	18	25.4	
		Severe	2	2.8	
**Length of hospitalization (days)**	295	Up to 1 day	31	10.5	**<0.001**
		2 days	98	33.2	
		3 days	94	31.9	
		4 days	31	10.5	
		≥5 days	41	13.9	
**Nutritional status**	295	Malnourished	30	10.2	**<0.001**
		Well-nourished	237	80.3	
		Overweight	28	9.5	
**Disease of diagnosis**	295	Acute gastroenteritis	97	32.9	**<0.001**
		Respiratory tract infection	83	28.1	
		Urinary tract infection	15	5.1	
		Asthma	7	2.4	
		Other diseases	93	31.5	

* Missing data.

**Table 2:** Difference between numbers of male and female patients with anemia enrolled in the study.

Age	Hgb Cut-off	Total		Males		Females		P-value
		N	%	n	%	n	%	
**1 month**	**<10.7**	2	2.5	1	50.0	1	50.0	0.809
**2 months**	**<9.4**	6	7.4	3	50.0	3	50.0	0.696
**3–5 months**	**<9.5**	2	2.5	1	50.0	1	50.0	1.000
**6–59 months**	**<11**	60	74.1	32	53.3	28	46.7	0.726
**≥60 months to <12 years**	**<11.5**	11	13.6	8	72.7	3	27.3	0.101

Hgb: hemoglobin.

**Table 3:** Severity of anemia categorized in children aged 6 months and above.

Age	Severity	Hgb range (g/dL)	Number	%	P-value
**6–59 months**	**Mild**	**10–10.9**	46	76.7	**0.002**
	**Moderate**	**7–9.9**	14	23.3	
	**Severe**	**<7**	0	0.0	
**≥60 months to <12 years**	**Mild**	**11–11.4**	5	45.5	**0.002**
	**Moderate**	**8–10.9**	4	36.4	
	**Severe**	**<8**	2	18.2	

Hgb: hemoglobin.

The weight-for-age measurement was used to assess the nutritional status. Weight is measured as a routine procedure on the first day of admission to the hospital. We used the weight z-score for analysis, based on WHO standards for classification: underweight for age (z-score < -2), adequate weight (z-score ≥ -2 and < +2), and overweight for age (z-score ≥ +2) [2].

### Statistical analysis

Statistical analyses were conducted using the Statistical Package for Social Science software (SPSS, Inc.), version 20.0, which was used also for data management and cleaning. Descriptive statistics was carried out and reported as number and percent for categorical variables, whereas the mean and standard deviation (±) for continuous ones. The WHO and AAP criterion were used to stratify patients into anemic and non-anemic groups. Chi-square test was used to assess any significant difference between two groups. Logistic regression was used to determine the associations between anemic cases (yes/no) as dependent variable and age (months), nutritional status, infectious disease, breast feeding, solid food introduction (before 6 months), meat intake (before 6 months) and iron supplement as independent variables. The level of significance was set at P < 0.05 for all statistical analyses. Data points behind means, medians and variance measures are available in **S1 Table**.

## Results

### Socio-demographic and clinical characteristics of patients

A total of 295 children patients were included in this cross-sectional clinical study. Summary of the clinical characteristics of the patients is shown in **Table 1**. The overall male-to-female ratio of the included cases was 1.18:1 (54.2% males and 45.8% females). The predominant age group ranged between 6 and 59 months (71.9%). The mean age of patients was 31.8 ± 29.2 months. Majority of children were well-nourished (80.3%) and 13.9% were hospitalized for five or more days. The most frequent diseases of diagnosis at admission included gastroenteritis (32.9%) and respiratory tract infections (28.1%). Among anemic patients, 71.8% and 25.4% of children admitted suffered from mild and moderate anemia, respectively. The majority of children were breast-fed for more than 4 months (50.9%) as well as given iron supplement (64%) (**Table 4**).

**Table 4:** Prevalence of anemia stratified according to gender, age, length of hospitalization, nutritional status, disease of diagnosis, and other associated factors.

Clinical Parameter	Total number of patients	Categories	N (%)	Anemia Status				
				Anemic		Non-anemic		P-value
				n	%	n	%	
Gender*	**292**	**Males**	158 (54.1)	45	28.5	113	71.5	0.759
		**Females**	134 (45.9)	36	26.9	98	73.1	
Age (months)*	**292**	**1 month**	7 (2.4)	2	28.6	5	71.4	0.960
		**2 months**	16 (5.5)	6	37.5	10	62.5	0.370
		**3–5 months**	12 (4.1)	2	16.7	10	83.3	0.382
		**6–59 months**	210 (71.9)	60	28.6	150	71.4	0.611
		**≥60 months to <12 years**	47 (16.1)	11	23.4	36	76.6	0.469
Length of hospitalization (days)*	**292**	**1 day**	30 (10.3)	9	30.0	21	70.0	0.770
		**2 days**	96 (32.9)	23	24.0	73	76.0	0.312
		**3 days**	94 (32.2)	24	25.5	70	74.5	0.562
		**4 days**	31 (10.6)	8	25.8	23	74.2	0.799
		**≥5 days**	41 (14.0)	17	41.5	24	58.5	**0.034**
Nutritional Status*	**292**	**Malnourished**	29 (9.9)	14	48.3	15	51.7	**0.009**
		**Well-nourished**	235 (80.5)	61	26.0	174	74.0	0.167
		**Overweight**	28 (9.6)	6	21.4	22	78.6	0.433
Disease of diagnosis*	**292**	**Acute gastroenteritis**	96 (32.9)	24	25.0	72	75.0	0.464
		**Respiratory tract infection**	83 (28.4)	23	27.7	60	72.3	0.994
		**Urinary tract infection**	15 (5.1)	5	33.3	10	66.7	0.619
		**Asthma**	7 (2.4)	2	28.6	5	71.4	0.960
		**Other diseases**	91 (31.2)	27	29.7	64	70.3	0.620
Breast feeding (months)*	**289**	**<1**	54 (18.7)	12	22.2	42	77.8	0.604
		**1–4**	88 (30.4)	25	28.4	63	71.6	
		**>4**	147 (50.9)	43	29.3	104	70.7	
Solid food introduction (months)*	**256**	**≤6**	207 (80.9)	54	26.1	153	73.9	0.354
		**>6**	49 (19.1)	16	32.7%	33	67.3	
Meat intake (months)*	**223**	**≤6**	88 (39.5)	19	21.6	69	78.4	0.119
		**>6**	135 (60.5)	42	31.1	93	68.9	
Iron supplement*	**264**	**Yes**	169 (64.0)	40	23.7	129	76.3	**0.054**
		**No**	95 (36.0)	33	34.7	62	65.3	

*Missing data.

### Stratification of anemia by age, gender, and severity

Anemia among patients was defined based on cutoff Hgb values specific to each age group, as per the WHO and AAP criteria into anemic and non-anemic groups (**Table 2**), and further categorized into mild, moderate, and severe in children aged 6 months and above as shown in **Table 3**. There was no significant difference between males and females regarding their anemia status.

### Prevalence of anemia and associated factors

Results of the chi-square test for trend indicated that the proportion of anemic cases is not affected by age, where no statistically significant difference between the anemic and non-anemic group of patients was found among each of the different age categories (**Table 4**). No significant difference between males and females was as well found (p-value = 0.759) noting that anemia affected 28.5% of males and 26.9% of females. Noteworthy, anemia was statistically related to iron supplementation, where percentage of anemia increases from 23.7 to 34.7% between anemic patients on iron and those who were not (p-value = 0.054).

Severe anemia has been associated with increased hospital length of stay [18]. Our results however showed that anemia is not correlated with length of hospitalization, which is explained by the lack of severe anemic cases among our population under study. In terms of clinical diagnosis, the chi-square test indicated that there was no statistically significant association with the anemia status. Comparisons between the remaining groups were not significant (**Table 4**).

Multivariate analysis revealed that risk of anemia increases by around 3.4 folds among malnourished children than in well-nourished children (PR = 3.422; p-value = 0.038). On the other hand, we have found an approximately 42% decrease in the risk of anemia among children on iron supplements (PR = 0.583; p-value = 0.055) (**Table 5**).

**Table 5:** Prevalence analysis adjusted for anemia among children.

Variable	Categories	PR*	95% CI	P-value
**Age (months)**	**1 month**	0.764	(0.130–4.500)	0.766
	**2 months**	0.509	(0.151–1.719)	0.277
	**3–5 months**	1.528	(0.290–8.048)	0.617
	**6–59 months**	0.764	(0.365–1.599)	0.475
	**≥60 months to <12 years**	1.0	-	-
**Nutritional Status**	**Malnourished**	3.422	(1.073–10.915)	**0.038**
	**Well-nourished**	1.0	-	-
	**Overweight**	0.778	(0.301–2.009)	0.604
**Infectious diagnosis**	**Acute gastroenteritis**	1.266	(0.664–2.412)	0.474
	**Respiratory tract infection**	1.101	(0.570–2.126)	0.775
	**Urinary tract infection**	0.844	(0.263–2.702)	0.775
	**Asthma**	1.055	(0.193–5.776)	0.951
	**Other diseases**	1.0	-	-
**Breast feeding**	**<1**	1.750	(0.146–20.996)	0.659
	**1–4**	1.260	(0.109–14.525)	0.853
	**>4**	1.0	-	-
**Solid food introduction**	**≤6**	1.374	(0.701–2.692)	0.355
	**>6**	1.0	-	-
**Meat intake**	**≤6**	1.640	(0.878–3.064)	0.121
	**>6**	1.0	-	-
**Iron supplement**	**Yes**	0.583	(0.336–1.011)	**0.055**
	**No**	1.0	-	-

*Prevalence Ratio.

### Characterization of children in relation to hemoglobin levels

**Fig 1:**
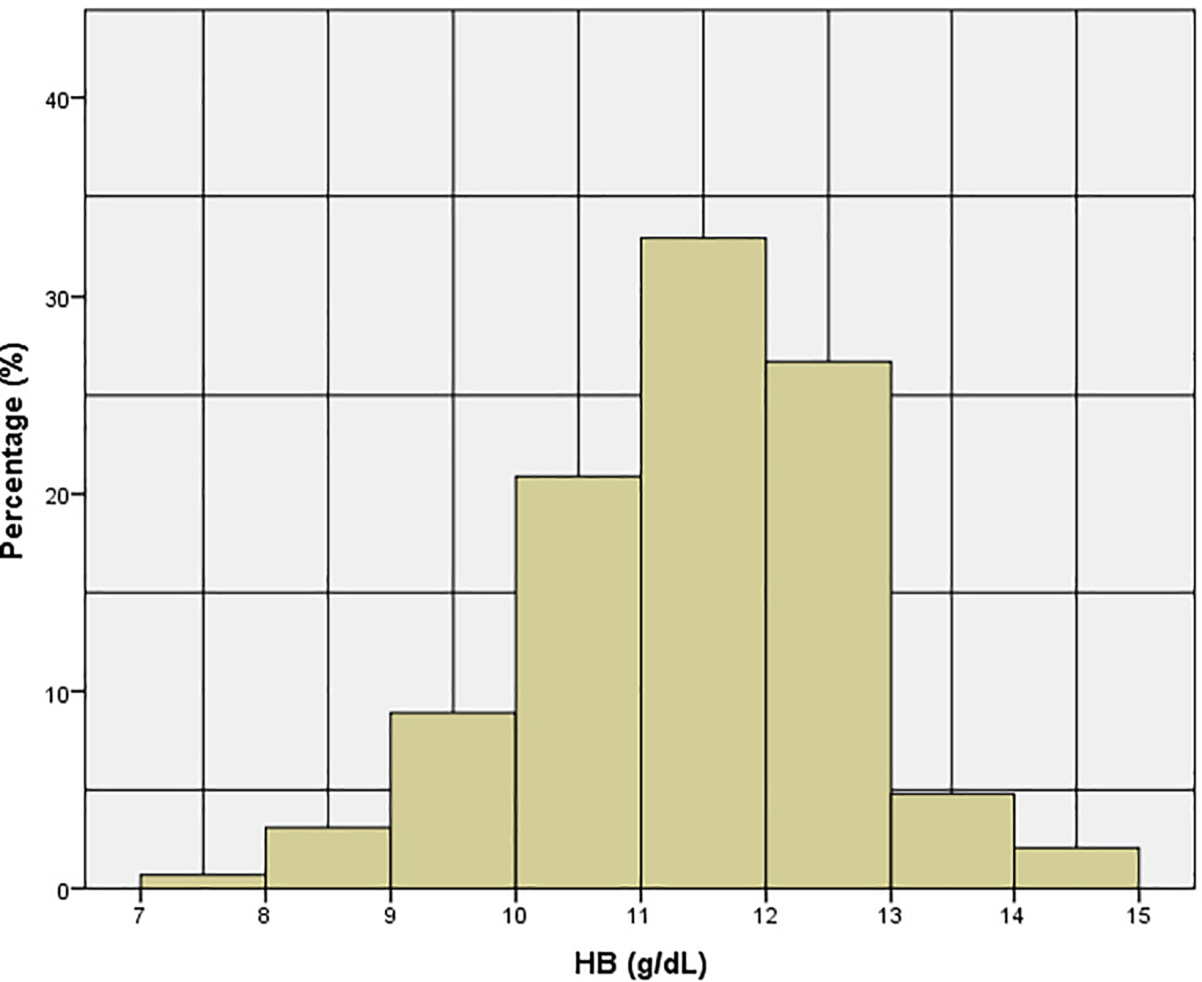
Distribution of hemoglobin concentration among children.

The distribution of hemoglobin levels is presented in “**Fig 1**”. Overall, there was a 33.2% prevalence of anemia (95% CI: 9.95–10.27) and the average hemoglobin content was 11.5 g/dL ± 1.2 g/dL. Moderate anemia was found in 12.5% (95% CI: 9.07–9.56) of children and the mildest form was found in 20.7% (95% CI: 10.53–10.67).

## Discussion

There is no study performed in Lebanon yet that analyzes prevalence and risk factors associated with anemia in hospitalized children. This study was performed in a tertiary care hospital located in a rural area of South Lebanon and included 295 hospitalized children admitted with different clinical diagnoses. The prevalence of anemia was found to be 33.2%, which is considered a moderate public health problem as it goes in line with the WHO severity classification of anemia that sets a threshold percentage of 40% for countries with severe anemia status such as in some African countries. In other developed countries like Australia, Canada and most of the European countries, incidence of anemia is less than 15% [19].

Regarding the relationship with sex, our results reveal merely close percentages of anemia in male and female patients (28.5% of males and 26.9% of females) without a significant statistical level; however, many authors reported that anemia is more prevalent in boys, a problem that may be due to the faster growth of pre-school boys compared to girls, prompting high iron requirement. Nonetheless, it is crucial to do more studies in order to better delineate gender factor relationship to anemia in this context [20].

In our study, higher prevalence of anemia was found in the age group of children 6–59 months (71.9%), which is close to the result reported by Neves *et al*. in 2005, where prevalence of anemia among children aged below 24 months was found to be 55.1% [21]. Those value are expected for this age group due to the increased iron demands during this rapid growth period, early weaning, and lack of foods rich in iron. Furthermore, unfavorable living conditions that render children more susceptible to develop diarrhea, respiratory infections and intestinal parasites may negatively affect the intake, absorption and biological utilization of iron [20].

Our results showed that the prevalence of anemia was positively correlated with the nutritional status, considering the z score of the weight-for-age of less than -2 standard deviation to be malnourished, where were found a 3.4 higher relative risk of having anemia among malnourished children than their comparable group of well-nourished patients. Our results are comparable to the study conducted by Rocha *et al*. that demonstrated means of z-score for height/age and weight/age being significantly lower in anemic children, compared to non-anemic ones [22].

It is difficult to interpret the correlation between anemia and the length of hospitalization as many other factors may play a significant role in this context, such as the underlying medical condition, different medical care and the effect of the sample randomization [23]. In our study, no significant correlation was found between the two variables.

In our study, anemia was most common in children suffering from acute gastroenteritis followed by respiratory tract infections. Generally speaking, respiratory tract infections require greater utilization of hemoglobin due to the infectious process itself and increased respiratory effort. On the other hand, gastrointestinal diseases contribute to loss of blood either in vomitus and feces or via degradation by parasites. In accordance, Lima *et al*. reported a higher prevalence of anemia in infants with infectious diarrhea [24], as reported in our study.

There are many causes of microcytic anemia including: thalassemia hemoglobinopathies, iron depletion, anemia of chronic diseases, and chronic lead intoxication. In attempt to clarify these possible etiologies, one needs to take into account the patient’s medical history, the RBC distribution width (RDW) and the mean corpuscular volume (MCV) which varies in reference to the age [25].

It is well established that early iron supplementation during the first year of rapid growth is a protective factor against anemia, unlike other dietary acts of solid food introduction or prolonged breast feeding that were found to have no effect on anemia prevention [26, 27].

The limitations of our study include: small sample size obtained, single-center study, short-period study (5-months cross-sectional study), and the absence of specific blood tests and other missing data in the medical records of patients, which disabled us from determining the etiology of anemia. In addition, we based our results on the first CBCD performed on admission due to lack of other consecutive blood tests.

## Conclusions

Anemia in children is a common preventable health issue in Lebanese children. Being more frequent in children below 12 months of age, it may predispose this vulnerable population to future hematologic, infectious, psychomotor and developmental disorders. These disorders can be primarily prevented by proper nutritional habits and good supplementation of essential factors involved in the production of red blood cells, such as early iron supplementation of both breast- and formula-fed infants during their first year of life, along with proper infant and young child feeding (IYCF) based on the introduction of iron-rich food, and by the prevention of malnutrition which is prevalent in 10% of our studied population.

## Supporting information

S1 Table Complete patients’ demographic and medical data underlying the findings described in the manuscript. (XLS)
